# (Benzyl phenyl sulfoxide-κ*O*)­chlorido­triphenyl­tin(IV)

**DOI:** 10.1107/S1600536812005910

**Published:** 2012-02-17

**Authors:** Guo-Xia Tan, Chang-Fa Zhang

**Affiliations:** a School of Chemistry and Chemical Engineering, Linyi University, Linyi 276000, People’s Republic of China; b Shandong Water Polytechnic, Rizhao 276826, People’s Republic of China

## Abstract

The Sn^IV^ atom in the title compound, [Sn(C_6_H_5_)_3_Cl(C_13_H_12_OS)], is situated within a distorted C_3_ClO trigonal–bipyramidal coordination geometry with a mean Sn—C distance of 2.136 (6) Å and with an Sn—O distance of 2.393 (4) Å. The Sn^IV^ atom lies 0.171 (3) Å out of the equatorial C_3_ plane in the direction of the axially bound Cl atom.

## Related literature

For background to the structures, biological activities and industrial applications of triorganotin(IV) complexes, see: Davies *et al.* (2008 ▶); Tian *et al.* (2005 ▶). For related organotin sulfoxide structures, see: Fuller *et al.* (2009 ▶); Kumar *et al.* (2009 ▶); Filgueiras *et al.* (1982 ▶); Dokorou *et al.* (2011 ▶).
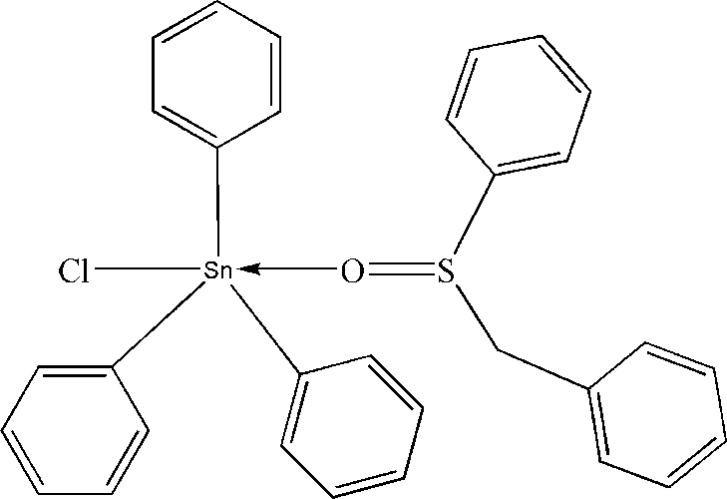


## Experimental

### 

#### Crystal data

[Sn(C_6_H_5_)_3_Cl(C_13_H_12_OS)]
*M* *_r_* = 601.73
Orthorhombic, 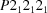

*a* = 9.744 (5) Å
*b* = 10.016 (3) Å
*c* = 28.840 (5) Å
*V* = 2814.7 (17) Å^3^
*Z* = 4
Mo *K*α radiation
μ = 1.10 mm^−1^
*T* = 295 K
0.32 × 0.28 × 0.21 mm

#### Data collection

Bruker P4 diffractometer
Absorption correction: ψ scan (*XSCANS*; Bruker, 1996 ▶) *T*_min_ = 0.720, *T*_max_ = 0.802
3690 measured reflections
3463 independent reflections
2794 reflections with *I* > 2σ(*I*)
*R*_int_ = 0.021
3 standard reflections every 97 reflections intensity decay: 2.3%

#### Refinement

*R*[*F* ^2^ > 2σ(*F* ^2^)] = 0.037
*wR*(*F* ^2^) = 0.067
*S* = 1.02
3463 reflections
306 parameters
H-atom parameters constrained
Δρ_max_ = 0.37 e Å^−3^
Δρ_min_ = −0.40 e Å^−3^
Absolute structure: Flack (1983 ▶), 626 Friedel pairs
Flack parameter: 0.02 (3)

### 

Data collection: *XSCANS* (Bruker, 1996 ▶); cell refinement: *XSCANS*; data reduction: *XSCANS*; program(s) used to solve structure: *SHELXS97* (Sheldrick, 2008 ▶); program(s) used to refine structure: *SHELXL97* (Sheldrick, 2008 ▶); molecular graphics: *ORTEP-3* (Farrugia, 1997 ▶); software used to prepare material for publication: *SHELXL97*.

## Supplementary Material

Crystal structure: contains datablock(s) global, I. DOI: 10.1107/S1600536812005910/tk5048sup1.cif

Structure factors: contains datablock(s) I. DOI: 10.1107/S1600536812005910/tk5048Isup2.hkl

Additional supplementary materials:  crystallographic information; 3D view; checkCIF report

## Figures and Tables

**Fig. 1.**
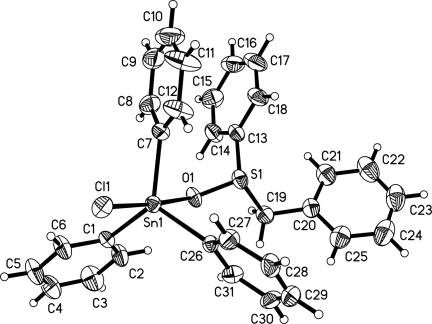
The molecular structure of (I) with displacement ellipsoids are drawn at the 30% probability level.

## supplementary crystallographic information

### Comment

Triorganotin compounds have received considerable attention due to their structural diversity and increasing numbers of industrial, agricultural and biological applications (Davies *et al.*, 2008; Tian *et al.*, 2005). Several structures of triorganotin sulfoxide complexes, such as chloro(dimethylsulfoxide)triphenyltin (Kumar *et al.*, 2009), 1,2-bis(*n*-propylsulfinyl)ethylene)bis(chlorotriphenyltin) (Filgueiras *et al.*, 1982), and (2-((2,3-dichlorophenyl)amino)benzoato)(dimethylsulfoxide)triphenyltin (Dokorou *et al.*, 2011), have been reported. As a continuation of these studies, the structure of the title compound, (I), is described herein.

The coordination environment of the tin^IV^ atom in (I) can be described as a distorted trigonal bipyramid with three phenyl groups occupying the equatorial positions whereas the axial positions are occupied by the Cl1 atom and the sulfoxide O1 atom (Fig. 1).The Sn atom is slightly displaced from the equatorial plane defined by the C_3_ set by 0.171 (3) Å in the direction of the Cl1 atom. The Sn—C and Sn—Cl bond lengths are similar to these found in chloro(dimethylsulfoxide-*κO*)triphenyltin (Kumar *et al.*, 2009). However, the Sn—O length (2.393 (4) Å) is longer than that in the above mentioned structure (2.310 (2) Å). The S═O bond length (1.524 (4) Å) is longer than that in the free ligand (1.500 (2) Å) (Fuller *et al.*, 2009) due to the O1 atom coordination to Sn atom. The dihedral angle between two phenyl rings in the sulfoxide ligand is 54.56 (5)°.

### Experimental

Benzylphenylsulfoxide (0.43 g, 2 mmol) and triphenyltin chloride (0.77 g, 2 mmol) in ethanol (40 ml) were refluxed for 1 h, and then the colourless solution was reduced to 15 ml under reduce pressure. The colourless crystals suitable for X-ray analysis were obtained by slow evaporation of the solution at room temperature (yield: 62%; *M*.pt.: 385–386 K).

### Refinement

H atoms were placed at calculated positions (C—H = 0.97 Å for methylene-H and C—H = 0.93 Å for aromatic-H atoms) and refined as riding with *U*_iso_(H) = 1.2*U*_eq_(C).

### Crystal data

**Table:**

[Sn(C_6_H_5_)_3_Cl(C_13_H_12_OS)]	*F*(000) = 1216
*M**_r_* = 601.73	*D*_x_ = 1.420 Mg m^−^^3^
Orthorhombic, *P*2_1_2_1_2_1_	Mo *K*α radiation, λ = 0.71069 Å
Hall symbol: P 2ac 2ab	Cell parameters from 35 reflections
*a* = 9.744 (5) Å	θ = 5.1–12.5°
*b* = 10.016 (3) Å	µ = 1.10 mm^−^^1^
*c* = 28.840 (5) Å	*T* = 295 K
*V* = 2814.7 (17) Å^3^	Block, colourless
*Z* = 4	0.32 × 0.28 × 0.21 mm

### Data collection

**Table:**

Bruker P4 diffractometer	2794 reflections with *I* > 2σ(*I*)
Radiation source: fine-focus sealed tube	*R*_int_ = 0.021
Graphite monochromator	θ_max_ = 25.0°, θ_min_ = 2.2°
ω scans	*h* = −1→11
Absorption correction: ψ scan (*XSCANS*; Bruker, 1996)	*k* = −1→11
*T*_min_ = 0.720, *T*_max_ = 0.802	*l* = −1→34
3690 measured reflections	3 standard reflections every 97 reflections
3463 independent reflections	intensity decay: 2.3%

### Refinement

**Table:**

Refinement on *F*^2^	Hydrogen site location: inferred from neighbouring sites
Least-squares matrix: full	H-atom parameters constrained
*R*[*F*^2^ > 2σ(*F*^2^)] = 0.037	*w* = 1/[σ^2^(*F*_o_^2^) + (0.024*P*)^2^ + 0.0502*P*] where *P* = (*F*_o_^2^ + 2*F*_c_^2^)/3
*wR*(*F*^2^) = 0.067	(Δ/σ)_max_ < 0.001
*S* = 1.02	Δρ_max_ = 0.37 e Å^−^^3^
3463 reflections	Δρ_min_ = −0.40 e Å^−^^3^
306 parameters	Extinction correction: *SHELXL97*, Fc^*^=kFc[1+0.001xFc^2^λ^3^/sin(2θ)]^-1/4^
0 restraints	Extinction coefficient: 0.00117 (16)
Primary atom site location: structure-invariant direct methods	Absolute structure: Flack (1983), 626 Friedel pairs
Secondary atom site location: difference Fourier map	Flack parameter: 0.02 (3)

### Special details

**Table:**

Geometry. All e.s.d.'s (except the e.s.d. in the dihedral angle between two l.s. planes) are estimated using the full covariance matrix. The cell e.s.d.'s are taken into account individually in the estimation of e.s.d.'s in distances, angles and torsion angles; correlations between e.s.d.'s in cell parameters are only used when they are defined by crystal symmetry. An approximate (isotropic) treatment of cell e.s.d.'s is used for estimating e.s.d.'s involving l.s. planes.
Refinement. Refinement of *F*^2^ against ALL reflections. The weighted *R*-factor *wR* and goodness of fit *S* are based on *F*^2^, conventional *R*-factors *R* are based on *F*, with *F* set to zero for negative *F*^2^. The threshold expression of *F*^2^ > σ(*F*^2^) is used only for calculating *R*-factors(gt) *etc*. and is not relevant to the choice of reflections for refinement. *R*-factors based on *F*^2^ are statistically about twice as large as those based on *F*, and *R*- factors based on ALL data will be even larger.

### Fractional atomic coordinates and isotropic or equivalent isotropic displacement parameters (Å^2^)

**Table:**

	*x*	*y*	*z*	*U*_iso_*/*U*_eq_	
Sn1	0.09780 (5)	0.65032 (4)	0.122403 (15)	0.04029 (13)	
O1	−0.1431 (4)	0.6938 (4)	0.12855 (16)	0.0497 (12)	
Cl1	0.34939 (15)	0.60290 (16)	0.12177 (6)	0.0534 (4)	
S1	−0.19603 (16)	0.83689 (17)	0.13031 (6)	0.0502 (4)	
C1	0.0329 (7)	0.4480 (6)	0.1119 (2)	0.0423 (17)	
C2	−0.0971 (9)	0.4170 (7)	0.0986 (2)	0.064 (2)	
H2	−0.1620	0.4846	0.0959	0.077*	
C3	−0.1346 (9)	0.2866 (8)	0.0890 (3)	0.086 (3)	
H3	−0.2243	0.2680	0.0800	0.103*	
C4	−0.0424 (9)	0.1856 (7)	0.0925 (3)	0.076 (3)	
H4	−0.0676	0.0985	0.0854	0.091*	
C5	0.0869 (9)	0.2137 (7)	0.1067 (3)	0.079 (3)	
H5	0.1504	0.1452	0.1101	0.095*	
C6	0.1254 (7)	0.3454 (7)	0.1162 (2)	0.066 (2)	
H6	0.2147	0.3636	0.1256	0.079*	
C7	0.1114 (6)	0.7524 (5)	0.18703 (12)	0.0494 (18)	
C8	0.2066 (6)	0.8553 (5)	0.19079 (17)	0.089 (3)	
H8	0.2597	0.8792	0.1653	0.107*	
C9	0.2224 (6)	0.9224 (5)	0.2326 (2)	0.122 (4)	
H9	0.2861	0.9912	0.2351	0.146*	
C10	0.1431 (7)	0.8867 (6)	0.27072 (15)	0.111 (4)	
H10	0.1536	0.9316	0.2987	0.133*	
C11	0.0479 (6)	0.7838 (6)	0.26696 (13)	0.098 (4)	
H11	−0.0052	0.7599	0.2924	0.117*	
C12	0.0321 (5)	0.7167 (5)	0.22511 (18)	0.070 (2)	
H12	−0.0316	0.6479	0.2226	0.084*	
C13	−0.2771 (7)	0.8539 (7)	0.1851 (2)	0.0495 (16)	
C14	−0.3607 (8)	0.7581 (7)	0.2023 (3)	0.066 (2)	
H14	−0.3769	0.6807	0.1853	0.079*	
C15	−0.4224 (9)	0.7757 (8)	0.2452 (3)	0.078 (2)	
H15	−0.4808	0.7106	0.2570	0.093*	
C16	−0.3965 (10)	0.8893 (9)	0.2701 (3)	0.087 (3)	
H16	−0.4384	0.9019	0.2988	0.105*	
C17	−0.3094 (9)	0.9846 (9)	0.2530 (3)	0.088 (3)	
H17	−0.2907	1.0608	0.2703	0.106*	
C18	−0.2498 (8)	0.9676 (7)	0.2103 (3)	0.066 (2)	
H18	−0.1912	1.0325	0.1985	0.079*	
C19	−0.3445 (6)	0.8314 (7)	0.0924 (2)	0.0564 (19)	
H19A	−0.3179	0.7908	0.0632	0.068*	
H19B	−0.4140	0.7750	0.1064	0.068*	
C20	−0.4055 (8)	0.9656 (7)	0.0828 (2)	0.0556 (17)	
C21	−0.4840 (8)	1.0320 (8)	0.1156 (3)	0.074 (2)	
H21A	−0.4991	0.9929	0.1444	0.088*	
C22	−0.5403 (9)	1.1564 (10)	0.1058 (4)	0.103 (3)	
H22A	−0.5896	1.2019	0.1285	0.124*	
C23	−0.5235 (11)	1.2117 (10)	0.0632 (5)	0.109 (4)	
H23A	−0.5649	1.2932	0.0566	0.131*	
C24	−0.4459 (10)	1.1491 (10)	0.0294 (4)	0.104 (4)	
H24A	−0.4332	1.1880	0.0004	0.125*	
C25	−0.3872 (9)	1.0254 (8)	0.0401 (3)	0.076 (2)	
H25A	−0.3344	0.9821	0.0178	0.091*	
C26	0.0997 (7)	0.7770 (6)	0.06277 (19)	0.0376 (14)	
C27	0.2015 (7)	0.8674 (7)	0.0545 (2)	0.0555 (19)	
H27	0.2750	0.8713	0.0750	0.067*	
C28	0.2003 (9)	0.9535 (7)	0.0170 (3)	0.067 (2)	
H28	0.2710	1.0146	0.0127	0.080*	
C29	0.0934 (11)	0.9471 (7)	−0.0136 (2)	0.069 (2)	
H29	0.0913	1.0040	−0.0391	0.082*	
C30	−0.0101 (8)	0.8575 (9)	−0.0068 (2)	0.068 (2)	
H30	−0.0821	0.8530	−0.0279	0.082*	
C31	−0.0087 (7)	0.7734 (7)	0.0310 (2)	0.0533 (19)	
H31	−0.0806	0.7137	0.0355	0.064*	

### Atomic displacement parameters (Å^2^)

**Table:**

	*U*^11^	*U*^22^	*U*^33^	*U*^12^	*U*^13^	*U*^23^
Sn1	0.0418 (2)	0.0370 (2)	0.0421 (2)	−0.0018 (3)	0.0005 (3)	0.0007 (2)
O1	0.038 (2)	0.037 (2)	0.074 (3)	0.0086 (19)	0.003 (2)	−0.006 (2)
Cl1	0.0402 (9)	0.0677 (10)	0.0523 (8)	0.0007 (8)	0.0055 (10)	0.0088 (10)
S1	0.0432 (9)	0.0374 (8)	0.0701 (11)	−0.0007 (9)	−0.0056 (9)	−0.0031 (11)
C1	0.044 (4)	0.034 (3)	0.049 (4)	0.002 (3)	0.004 (3)	−0.004 (3)
C2	0.051 (5)	0.046 (4)	0.094 (5)	0.007 (5)	−0.017 (5)	−0.009 (4)
C3	0.063 (6)	0.069 (5)	0.125 (7)	−0.018 (5)	−0.013 (5)	−0.014 (6)
C4	0.081 (6)	0.040 (5)	0.107 (6)	−0.012 (5)	0.012 (5)	−0.007 (4)
C5	0.067 (5)	0.044 (4)	0.126 (8)	0.008 (5)	−0.004 (6)	−0.012 (4)
C6	0.051 (4)	0.046 (3)	0.100 (5)	0.009 (4)	0.000 (5)	−0.011 (6)
C7	0.054 (5)	0.047 (4)	0.047 (4)	0.011 (5)	−0.001 (4)	−0.004 (3)
C8	0.105 (7)	0.083 (6)	0.080 (5)	−0.021 (7)	0.011 (5)	−0.042 (6)
C9	0.131 (9)	0.124 (9)	0.111 (8)	−0.033 (8)	0.008 (8)	−0.075 (7)
C10	0.120 (10)	0.144 (10)	0.068 (6)	0.055 (8)	−0.017 (6)	−0.043 (7)
C11	0.134 (10)	0.112 (7)	0.047 (5)	0.058 (7)	0.006 (5)	−0.005 (5)
C12	0.085 (6)	0.077 (5)	0.048 (4)	0.013 (5)	0.010 (4)	0.009 (4)
C13	0.047 (4)	0.041 (4)	0.060 (4)	0.006 (4)	−0.004 (4)	−0.009 (4)
C14	0.079 (6)	0.046 (4)	0.073 (5)	−0.002 (5)	0.010 (5)	−0.009 (4)
C15	0.078 (7)	0.090 (6)	0.066 (5)	−0.011 (6)	0.008 (5)	0.008 (5)
C16	0.082 (6)	0.115 (8)	0.065 (5)	0.022 (7)	−0.009 (6)	−0.029 (6)
C17	0.089 (8)	0.077 (6)	0.099 (7)	0.006 (6)	−0.017 (6)	−0.044 (6)
C18	0.060 (6)	0.061 (5)	0.077 (5)	−0.004 (5)	−0.009 (5)	−0.024 (5)
C19	0.050 (4)	0.054 (5)	0.065 (4)	0.003 (4)	−0.008 (4)	−0.009 (4)
C20	0.043 (4)	0.053 (4)	0.071 (4)	−0.003 (5)	−0.016 (5)	−0.001 (4)
C21	0.054 (5)	0.071 (5)	0.096 (6)	0.010 (5)	−0.019 (5)	−0.007 (6)
C22	0.072 (6)	0.075 (6)	0.163 (10)	0.024 (6)	−0.038 (7)	−0.020 (7)
C23	0.081 (8)	0.061 (6)	0.185 (13)	0.000 (6)	−0.077 (9)	0.020 (7)
C24	0.098 (9)	0.090 (7)	0.125 (8)	−0.016 (8)	−0.038 (7)	0.047 (7)
C25	0.068 (6)	0.080 (6)	0.080 (5)	−0.012 (6)	−0.017 (5)	0.011 (5)
C26	0.034 (3)	0.037 (3)	0.042 (3)	0.001 (4)	−0.001 (4)	0.001 (3)
C27	0.055 (5)	0.056 (5)	0.055 (4)	−0.008 (5)	−0.006 (4)	0.001 (4)
C28	0.086 (6)	0.044 (4)	0.070 (5)	−0.004 (5)	0.014 (5)	0.011 (4)
C29	0.098 (6)	0.056 (4)	0.051 (4)	0.004 (6)	0.013 (6)	0.011 (4)
C30	0.076 (5)	0.087 (6)	0.042 (4)	0.026 (6)	−0.013 (4)	−0.002 (5)
C31	0.053 (4)	0.061 (5)	0.046 (4)	−0.008 (4)	0.000 (4)	−0.003 (4)

### Geometric parameters (Å, º)

**Table:**

Sn1—C7	2.130 (3)	C14—H14	0.9300
Sn1—C26	2.138 (5)	C15—C16	1.369 (10)
Sn1—C1	2.145 (6)	C15—H15	0.9300
Sn1—O1	2.394 (4)	C16—C17	1.369 (11)
Sn1—Cl1	2.4972 (19)	C16—H16	0.9300
O1—S1	1.524 (4)	C17—C18	1.371 (10)
S1—C13	1.774 (6)	C17—H17	0.9300
S1—C19	1.815 (6)	C18—H18	0.9300
C1—C2	1.359 (9)	C19—C20	1.496 (9)
C1—C6	1.373 (8)	C19—H19A	0.9700
C2—C3	1.384 (9)	C19—H19B	0.9700
C2—H2	0.9300	C20—C25	1.380 (9)
C3—C4	1.357 (10)	C20—C21	1.386 (9)
C3—H3	0.9300	C21—C22	1.391 (10)
C4—C5	1.354 (10)	C21—H21A	0.9300
C4—H4	0.9300	C22—C23	1.358 (12)
C5—C6	1.398 (9)	C22—H22A	0.9300
C5—H5	0.9300	C23—C24	1.384 (13)
C6—H6	0.9300	C23—H23A	0.9300
C7—C8	1.3900	C24—C25	1.399 (11)
C7—C12	1.3900	C24—H24A	0.9300
C8—C9	1.3900	C25—H25A	0.9300
C8—H8	0.9300	C26—C27	1.364 (8)
C9—C10	1.3900	C26—C31	1.398 (8)
C9—H9	0.9300	C27—C28	1.382 (9)
C10—C11	1.3900	C27—H27	0.9300
C10—H10	0.9300	C28—C29	1.367 (11)
C11—C12	1.3900	C28—H28	0.9300
C11—H11	0.9300	C29—C30	1.364 (10)
C12—H12	0.9300	C29—H29	0.9300
C13—C14	1.353 (9)	C30—C31	1.379 (9)
C13—C18	1.378 (9)	C30—H30	0.9300
C14—C15	1.388 (9)	C31—H31	0.9300
			
C7—Sn1—C26	114.73 (19)	C15—C14—H14	120.1
C7—Sn1—C1	126.6 (2)	C16—C15—C14	119.6 (8)
C26—Sn1—C1	116.7 (2)	C16—C15—H15	120.2
C7—Sn1—O1	84.78 (19)	C14—C15—H15	120.2
C26—Sn1—O1	87.7 (2)	C17—C16—C15	120.3 (9)
C1—Sn1—O1	83.87 (19)	C17—C16—H16	119.8
C7—Sn1—Cl1	92.09 (16)	C15—C16—H16	119.8
C26—Sn1—Cl1	95.67 (19)	C16—C17—C18	120.0 (8)
C1—Sn1—Cl1	96.24 (18)	C16—C17—H17	120.0
O1—Sn1—Cl1	176.15 (12)	C18—C17—H17	120.0
S1—O1—Sn1	120.4 (2)	C17—C18—C13	119.7 (8)
O1—S1—C13	105.7 (3)	C17—C18—H18	120.2
O1—S1—C19	102.8 (3)	C13—C18—H18	120.2
C13—S1—C19	100.7 (3)	C20—C19—S1	113.6 (5)
C2—C1—C6	117.8 (6)	C20—C19—H19A	108.8
C2—C1—Sn1	122.0 (5)	S1—C19—H19A	108.8
C6—C1—Sn1	120.1 (5)	C20—C19—H19B	108.8
C1—C2—C3	121.2 (7)	S1—C19—H19B	108.8
C1—C2—H2	119.4	H19A—C19—H19B	107.7
C3—C2—H2	119.4	C25—C20—C21	118.2 (7)
C4—C3—C2	120.9 (8)	C25—C20—C19	120.2 (8)
C4—C3—H3	119.5	C21—C20—C19	121.6 (7)
C2—C3—H3	119.5	C20—C21—C22	120.6 (9)
C5—C4—C3	118.9 (7)	C20—C21—H21A	119.7
C5—C4—H4	120.5	C22—C21—H21A	119.7
C3—C4—H4	120.5	C23—C22—C21	120.1 (10)
C4—C5—C6	120.3 (7)	C23—C22—H22A	120.0
C4—C5—H5	119.8	C21—C22—H22A	120.0
C6—C5—H5	119.8	C22—C23—C24	121.3 (10)
C1—C6—C5	120.8 (7)	C22—C23—H23A	119.4
C1—C6—H6	119.6	C24—C23—H23A	119.4
C5—C6—H6	119.6	C23—C24—C25	117.9 (10)
C8—C7—C12	120.0	C23—C24—H24A	121.0
C8—C7—Sn1	117.8 (3)	C25—C24—H24A	121.0
C12—C7—Sn1	122.2 (3)	C20—C25—C24	121.9 (9)
C7—C8—C9	120.0	C20—C25—H25A	119.0
C7—C8—H8	120.0	C24—C25—H25A	119.0
C9—C8—H8	120.0	C27—C26—C31	116.9 (6)
C10—C9—C8	120.0	C27—C26—Sn1	122.8 (5)
C10—C9—H9	120.0	C31—C26—Sn1	120.3 (5)
C8—C9—H9	120.0	C26—C27—C28	123.0 (7)
C9—C10—C11	120.0	C26—C27—H27	118.5
C9—C10—H10	120.0	C28—C27—H27	118.5
C11—C10—H10	120.0	C29—C28—C27	118.8 (8)
C10—C11—C12	120.0	C29—C28—H28	120.6
C10—C11—H11	120.0	C27—C28—H28	120.6
C12—C11—H11	120.0	C30—C29—C28	120.1 (7)
C11—C12—C7	120.0	C30—C29—H29	119.9
C11—C12—H12	120.0	C28—C29—H29	119.9
C7—C12—H12	120.0	C29—C30—C31	120.5 (7)
C14—C13—C18	120.6 (6)	C29—C30—H30	119.8
C14—C13—S1	121.7 (5)	C31—C30—H30	119.8
C18—C13—S1	117.7 (6)	C30—C31—C26	120.7 (7)
C13—C14—C15	119.8 (7)	C30—C31—H31	119.7
C13—C14—H14	120.1	C26—C31—H31	119.7
